# 

**Published:** 1884-11

**Authors:** 


					﻿NOVEMBER, 1884.
OLD SERIES
Vol. XXV

NEW SERIES
Vol. I -No. 9. i

fflEDIG AH
JdurnaF

Edited
by
1 W.F.WESTMORELAND,^
H.V.M.MILLER.M.D.LL ,D.j
JAMES A.GRAY, M.D
Published By james p.harrison&co^
y	Atlanta, ga. /ag
SUBSCRIPTION: 2.00 A YEAR.
				

